# Sex and APOE genotype differences related to statin use in the aging population

**DOI:** 10.1002/trc2.12156

**Published:** 2021-05-02

**Authors:** Arianna Dagliati, Niels Peek, Roberta Diaz Brinton, Nophar Geifman

**Affiliations:** ^1^ Centre for Health Informatics University of Manchester Manchester UK; ^2^ The Manchester Molecular Pathology Innovation Centre University of Manchester Manchester UK; ^3^ Department of Electrical Computer and Biomedical Engineering University of Pavia Pavia Italy; ^4^ NIHR Manchester Biomedical Research Centre Manchester Academic Health Science Centre University of Manchester Manchester UK; ^5^ Department of Pharmacology College of Medicine University of Arizona Tucson Arizona USA; ^6^ Department of Neurology College of Medicine University of Arizona Tucson Arizona USA; ^7^ Center for Innovation in Brain Science University of Arizona Tucson Arizona USA

**Keywords:** aging population, Alzheimer's disease, *APOE* genotype, statins, UK Biobank

## Abstract

**Background:**

Significant evidence suggests that the cholesterol‐lowering statins can affect cognitive function and reduce the risk for Alzheimer's disease (AD) and dementia. These potential effects may be constrained by specific combinations of an individual's sex and apolipoprotein E (*APOE*) genotype.

**Methods:**

Here we examine data from 252,327 UK Biobank participants, aged 55 or over, and compare the effects of statin use in males and females. We assessed difference in statin treatments taking a matched cohort approach, and identified key stratifiers using regression models and conditional inference trees. Using statistical modeling, we further evaluated the effect of statins on survival, cognitive decline over time, and on AD prevalence.

**Results:**

We identified that in the selected population, males were older, had a higher level of education, better cognitive scores, higher incidence of cardiovascular and metabolic diseases, and a higher rate of statin use. We observed that males and those participants with an *APOE* ε4–positive genotype had higher probabilities of being treated with statins; while participants with an AD diagnosis had slightly lower probabilities. We found that use of statins was not significantly associated with overall higher rates of survival. However, when considering the interaction of statin use with sex, the results suggest higher survival rates in males treated with statins. Finally, examination of cognitive function indicates a potential beneficial effect of statins that is selective for *APOE* ε4–positive genotypes.

**Discussion:**

Our evaluation of the aging population in a large cohort from the UK Biobank confirms sex and *APOE* genotype as fundamental risk stratifiers for AD and cognitive function, furthermore it extends them to the specific area of statin use, clarifying their specific interactions with treatments.

## 1 BACKGROUND

Population aging has been recognized as a key policy issue worldwide. The proportion and absolute number of older people are increasing dramatically: by 2040, nearly one in seven people is projected to be aged over 75 years.^1^ Projections suggest there will be 66.1 million people aged 80 years and over in the European Union by 2080.^2^ These trends will have a major impact on public spending. In the UK, the Office for Budget Responsibility forecasts total spending to increase from 33.6% to 37.8% of gross domestic product (GDP) between 2019 and 2064—equivalent to current £79 billion—due mainly to the aging population.^3^ In the United States, Medicare expenditures are projected to rise to 6% to 9% of GDP with a predicted strain on federal budget and the national economy.^4^ The burden of these expenditures will mainly affect health‐care systems as much of health‐care expenditures are incurred in the last years of life. A crucial point for policy development is whether extended life span is matched by health span.

Healthy aging has been defined as “the process of developing and maintaining the functional ability that enables well‐being in older age.”^1^ It focuses on the perspective of elderly peoples’ trajectory of functioning rather than the only disease they are experiencing at a single point in time, and it includes the concept of intrinsic capacity, which is the composite of all the physical and mental capacities of an individual. Healthy life expectancy, which indicates a reduction of years spent in ill health, is not keeping pace with increasing life expectancy. This suggests an increasing prevalence of chronic age‐related conditions with long‐duration preclinical phases such as Alzheimer's disease (AD).^3^

**RESEARCH IN CONTEXT**

1. **Systematic review**: The authors reviewed the literature using traditional PubMed and Scopus sources and meeting abstracts and presentations. Literature evidence suggests that the use of statins can affect cognitive function and reduce the risk for Alzheimer's disease (AD) and dementia. Potential effects may also be constrained by specific combinations of an individual's sex and apolipoprotein E (*APOE*) genotype. We investigate statins’ effect on an aging population using the UK Biobank.
2. **Interpretation**: Our study describes how males and *APOE* ε4–positive genotype had higher probabilities of being treated with statins. Analyses stratified by sex and *APOE* genotypes shows higher survival rates in males treated with statins. A potential beneficial effect of statins on cognitive function was observed in *APOE* ε4–positive genotypes.
3. **Future directions**: Sex and *APOE* genotype are essential risk stratifiers for AD and cognitive function future studies. In the area of statin use, their interactions with treatments should be assessed and taken into consideration both for further investigation and clinical evaluations.

Sex differences in longevity are documented and feature in many species in addition to humans.^5^, ^6^, ^7^ While it is common for women to live longer than men, the magnitude of the difference in longevity differs across cultures and is modifiable by environmental factors; the difference in life span is declining in developed nations.^8^

Cholesterol metabolism has been shown to have an important role in age‐related disease such as AD^9^, ^10^ and mounting evidence suggests that statins, a class of cholesterol‐lowering drugs, may effect cognitive function and risk for older age–associated AD and dementia.^11^, ^12^, ^13^, ^14^, ^15^, ^16^, ^17^ Clinical trials evaluating the effects of statins in patients diagnosed with AD have failed to meet primary outcomes, resulting in no significant therapeutic benefit.^18^, ^19^, ^20^, ^21^ However, medical bioinformatic analyses conducted over the past 5 years indicate that statin therapies are associated with reduced risk of AD.^11^, ^22^ Recent studies investigating the benefits of statins on neurological outcomes suggest that when statins are prescribed for population at risk of age‐related diseases, they are associated with decreased incidence of AD, dementia, Parkinson's disease, multiple sclerosis, and amyotrophic lateral sclerosis.^23^ The interaction of genotypes of apolipoprotein E (*APOE*), a risk factor for AD involved in cholesterol metabolism, with statins’ pharmacodynamics and pharmacokinetics has been largely investigated,^24^, ^25^, ^26^ indicating a significant effect of the genetic polymorphisms on treatment responses in term of plasma lipid profile^27^, ^28^ and a strong association with the risk and the course of coronary heart diseases.^29^, ^30^ Additionally evidence suggests that variants of *APOE*, protective against risk of AD, also slowed cognitive decline.^31^ Sex differences, as well as the effects of the *APOE* genotype, are well documented in statin drug response.^32^, ^33^, ^34^ In a recent examination of the association between statin use and the incidence of AD, it was found that reduction in AD risk varied across statin molecules, sex, and race/ethnicity.^35^

A major resource to enable investigations in aging populations is the UK Biobank,^36^ aimed at improving the prevention, diagnosis, and treatment of a wide range of serious and life‐threatening diseases. The UK Biobank recruited 500,000 people aged between 40 and 69 years (with more than 200,000 of these over the age of 60) from across the United Kingdom. All subjects have provided extensive demographic and health‐related information as well as biologic samples and are continually followed. UK Biobank is linking to a wide range of electronic health records such as death, hospital episodes, and general practice.

The aims of our study are (1) to assess differences in treatments in the aging population and identify potential stratifiers for greater beneficial effects of statins; and (2) to evaluate the effect of statin use in the aging population on survival, AD incidence, and cognitive decline.

**FIGURE 1:**
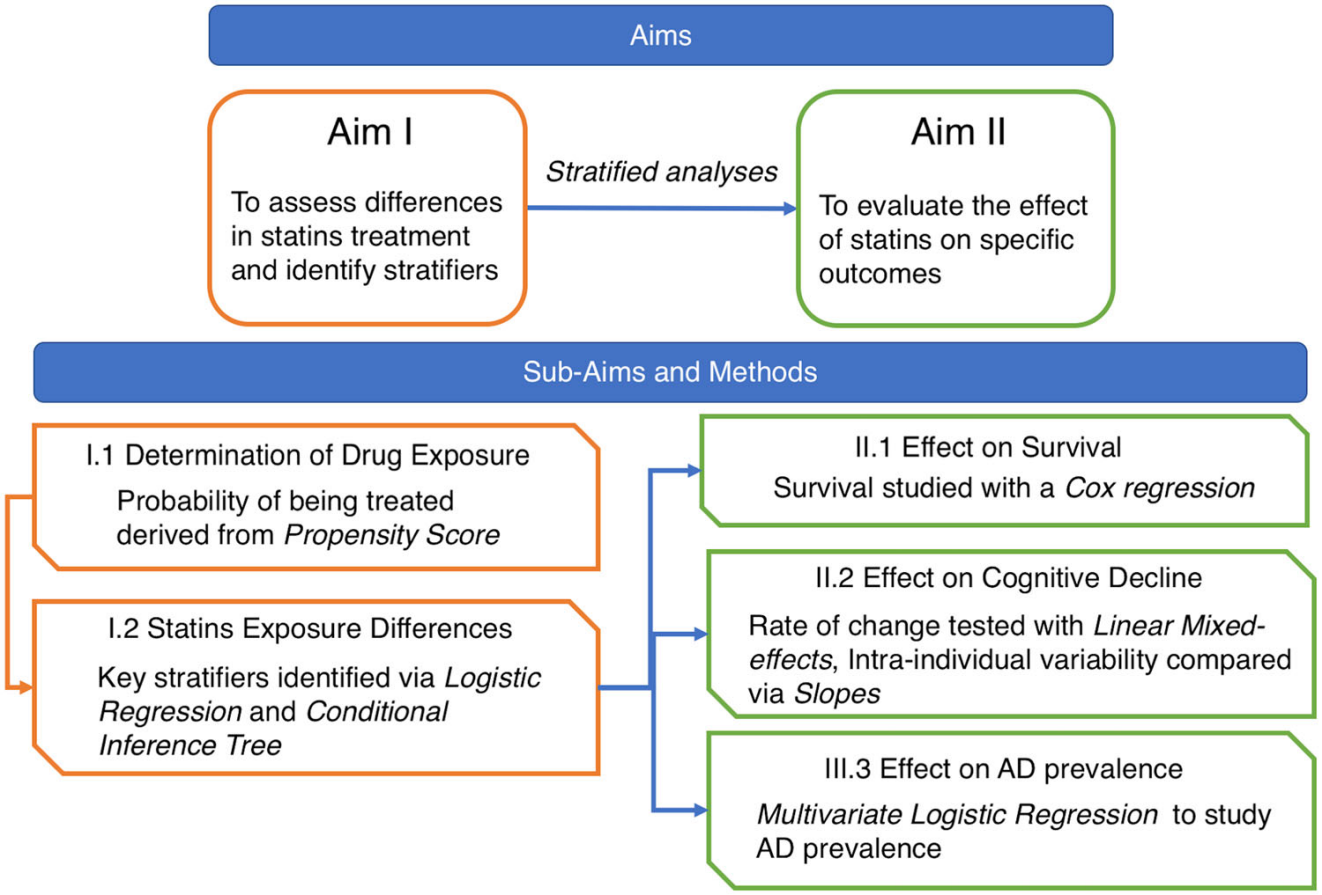
Study aims and analysis flow chart. The main aims are illustrated in the top boxes, subaims and their implementation in the bottom. Aim I (orange) is to assess differences in treatments in the aging population and identify potential stratifiers for greater beneficial effects of statins, to achieve the aim we determined drug exposure and assess their differences, focusing on statins treatments. Aim II (green) is to evaluate the potential beneficial effects of statin use in the aging population on survival, Alzheimer's disease (AD) incidence and cognitive decline; to achieve the aim we used the matched cohort and the identified stratifiers, derived from Aim I

While previously the cost‐effectiveness of a polypill, including simvastatin, to prevent cardiovascular diseases has been assessed in the UK Biobank cohort,^37^ to the best of our knowledge our work is the first to report on statin use within the UK Biobank's aging population. Study design and analytical strategy is described in Figure 1.

## 2 METHODS

### 2.1 Population and data

From the entire UK Biobank cohort, individuals aged 55 and over at recruitment (baseline) with determined *APOE* genotype were selected.

Baseline variables (age at recruitment, sex) were captured from the UK Biobank database. Social‐economic status of participants was assessed using the Townsend deprivation index, and participants were assigned an index score corresponding to the output area in which their postcode is located. Individuals’ education level was extracted as a binary variable from the education qualifications data and indicates if individuals have or do not have a college or university degree.^38^

### 2.2 Cognitive measures

Extensive descriptions of the cognitive function tests administered to UK Biobank participants—including missingness and intra‐variability over time—are provided by Lyall et al.^39^ From these scores, the Reaction Time (RT) test is used to assess reaction time, based on 12 rounds of the card‐game “Snap.”^40^ Participant are shown two cards at a time; if both cards are the same, they are instructed to press a button‐box as quickly as possible. The score on this task is the average response time in milliseconds across the 12 rounds.

Longitudinal studies of cognitive measures based on the UK Biobank are rare. A recent study^41^ used the RT to assess cognitive decline relationship with alcohol consumption. As shown in Lyall et al.,^39^ RT showed good stability across time points and higher reliability than other cognitive scores available in the UK Biobank.

Different from these previous studies, which used measurements at the baseline and at the first follow‐up, we use three time points: baseline and two follow‐up visits. Similar to previously demonstrated patterns, RT measures worsened over time, while Pair Test and Fluid Intelligence scores improved. Because RT scores were available for the largest number of UK Biobank participants at the three time points, we used this score as proxy for cognitive impairment. Higher RT values reflect an increase in cognitive impairment.

### 2.3 Disease diagnoses

Referring to the work in Zissimopoulos et al.,^35^ we included the diagnoses of the following prevalent conditions: AD, dementia, cardiovascular (acute myocardial infraction, atrial fibrillation, acute cerebrovascular disease, coronary atherosclerosis, angina, and hypertension) and metabolic diseases (diabetes, disorders of lipid metabolism). We used the International Classification of Diseases, Tenth Revision, Clinical Modification (ICD‐10‐CM) codes and applied the disease Clinical Classifications Software (CSS)^42^ on diagnosis codes to aggregate them into single‐level categories. Single, multilevel categories, and code sets are provided in File S1 in supporting information.

### 2.4 Determination of drug exposure

The medications category of the UK Biobank contains data on type and number of regular treatments taken by each individual. Data are obtained through a verbal interview by a trained nurse and coded via Read codes. We built a code set (reported in Appendix A in supporting information) for each of the medication groups of interest previously linked to cognitive impairment and included: statins, non‐statin cholesterol‐lowering drugs, AD medications, antidepressants, non‐steroidal anti‐inflammatory drugs (NSAIDs), estrogens, diabetes medications, vitamin E, omega‐3 and derivatives, and medications for long‐term asthma management.

### 2.5 Determination of *APOE* genotype

In the UK Biobank the *APOE* genotype is directly genotyped via SNPs rs429358 and rs7412. Values for either of the two SNPs were available for 299,627 participants; of these 47,299 participants were missing a value for one of the two SNPs and were therefore excluded. A total of 252,327 participants were included. *APOE* genotype missingness is due to UK Biobank enrollment procedures (i.e., participants recently enrolled for which the information is not available yet) or technical issues, therefore we assume are missing at random.^43^, ^44^ Further consideration regarding missingness mechanisms are reported in Appendix C in supporting information.

### 2.6 Statistical analyses

We compared and contrasted the population stratified by sex and *APOE* ε4 genotype. To test for significant differences among the four groups (female *APOE* ε4, female non‐*APOE* ε4, male *APOE* ε4, male non‐*APOE* ε4) we applied the Kruskal‐Wallis test for continuous variables, and chi‐square for categorical ones.

We compared *APOE* ε4 carriers within females and males using *t* tests and chi‐square. The Cochran‐Mantel‐Haenszel test was used for stratified analyses considering population distributions in ethnicity strata. Analyses of baseline differences in cohort characteristics were corrected for multiple testing using the Bonferroni correction, as indicated in Table 1.

**TABLE 1:** For comparison among four groups (female and male with/without *APOE* ε4) Kruskal‐Wallis and chi‐square tests were applied to test for significance

Sex	Female		*P*‐val	Male		*P*‐val	*P*‐val
*APOE* ε4 carrier	NO	YES	*APOE* ε4 in female (YES/NO)	NO	YES	APOE4 in male (YES/NO)	Comparing gender and APOE carrier
Number of patients	101366	35299		85763	29899		.905
Ethnicity (%)							
Asian	1507 (1.5)	282 (0.8)		1751 (2.0)	384 (1.3)		.065
Black	944 (0.9)	471 (1.3)		692 (0.8)	361 (1.2)		.604
Chinese	308 (0.3)	59 (0.2)		173 (0.2)	33 (0.1)		.985
Mixed	418 (0.4)	151 (0.4)		288 (0.3)	100 (0.3)		.791
Other ethnic group	754 (0.7)	201 (0.6)		538 (0.6)	139 (0.5)		.801
Not known	316 (0.3)	110 (0.3)		424 (0.5)	163 (0.5)		.490
White	97120 (95.8)	34025 (96.4)		81897 (95.5)	28719 (96.1)		.919
Mean age at recruitment (SD)	61.93 (4.1)	61.9 (4)	.206	62.22 (4.1)	62.24 (4.1)	.344	<. 0007^*^
University/college degree: YES (%)	40586 (40)	9377 (26.6)	.007	40763(47.5)	9465(31.7)	.084	<. 0007^*^
Townsend deprivation index (SD)	−1.55 (2.9)	−1.6 (2.9)	.004	−1.52(3)	−1.51(3)	.701	.645
Cognitive measures (SD)							
Fluid Intelligence	5.81 (2.1)	5.83 (2)	.261	6.09 (2.2)	6.08 (2.2)	.623	<. 0007^*^
Paris test–1st round	0.65 (1.3)	0.66 (1.3)	.356	0.57 (1.3)	0.58 (1.3)	.089	<.0007^*^
Paris test–2nd round	4.46 (3.4)	4.48 (3.5)	.151	4.51 (3.7)	4.45 (3.6)	.021	<.0007^*^
Reaction test	590.86 (122.3)	589 (119.2)	.019	570.07 (119.5)	572.24 (120.9)	.003	<.0007^*^
Diagnoses (%)							
AD dementia	110 (0.1)	116 (0.3)	<.0007^*^	112 (0.1)	106 (0.4)	<. 0007^*^	.192
Acute myocardial infraction	220 (0.2)	154 (0.4)	<.0007^*^	397 (0.5)	175 (0.6)	<. 0007^*^	<.0007^*^
Atrial fibrillation	1578 (1.6)	444 (1.3)	<.0007^*^	5450 (6.4)	1449 (4.8)	<. 0007^*^	<.0007^*^
Hypertension	5717 (5.6)	1381 (3.9)	.144	10323 (12)	2476 (8.3)	.073	<.0007^*^
Diabetes	32939 (32.5)	7685 (21.8)	.441	36681 (42.8)	8815 (29.5)	<. 0007^*^	<.0007^*^
Acute cerebrovascular	7009 (6.9)	1492 (4.2)	.003	11025 (12.9)	2375 (7.9)	.002	<.0007^*^
Disease	1056 (1)	233 (0.7)	.537	1760 (2.1)	460 (1.5)	.018	<.0007^*^
Coronary atherosclerosis	6651 (6.6)	1675 (4.7)	.002	16684 (19.5)	4339 (14.5)	<. 0007^*^	<.0007^*^
Disorders lipid metabolism	12057 (11.9)	3616 (10.2)	<.0007^*^	19270 (22.5)	5333 (17.8)	<. 0007^*^	<.0007^*^
Angina	5845(5.8)	1502 (4.3)	<.0007^*^	10855 (12.7)	2849 (9.5)	<. 0007^*^	<.0007^*^
Statin use (%)	22376 (22.1)	6728 (19.1)	<.0007^*^	36020 (42)	9851 (32.9)	<.0007^*^	<.0007^*^
Simvastatin use	16180 (16)	4665 (13.2)	<.0007^*^	25858 (30.2)	6777 (22.7)	<.0007^*^	<.0007^*^
Atorvastatin use	3913 (3.9)	1281 (3.6)	<.0007^*^	6400 (7.5)	2003 (6.7)	<.0007^*^	<.0007^*^
Pravastatin use	716 (0.7)	224 (0.6)	<.0007^*^	1135 (1.3)	295 (1)	.072	<.0007^*^
Rosuvastatin use	844 (0.8)	325 (0.9)	<.0007^*^	1110 (1.3)	397 (1.3)	<.0007^*^	<.0007^*^

For the comparison of *APOE* ε4 carriers within females and males *t* test and and chi‐square were used. We corrected the results for multiple testing using alpha = 0.05/66 = 0.00076, where 66 is the number of test performed.

Ns *P*‐val > .0007, ^*^ *P*‐val < = .0007.

Abbreviations: AD, Alzheimer's disease; APOE, apolipoprotein E; SD, standard deviation.

All analyses were computed using R version 3.2.3. Results are presented as the main effect with a 95% confidence interval. A significance level of 5% was used for main inferences.

To study drug exposure, while minimizing the effects of possible confounders and including relevant stratifiers, we applied propensity scoring to assess the comparability of case mix and created matched data sets for each drug category.

Given the definition of propensity scores (PS; i.e., the probability of being treated) this step allows us to compare the score in females and males, thus assessing relevant differences in treatments between sexes. To adjust for different distributions of characteristics across treated groups (age, social‐economic status, education level, and relevant diagnoses for each drug), patients were stratified based on their propensity of being treated with a specific drug. It is important to note that sex is not included as a potential confounder, as the aim of this analysis was to study its correlation with treatments, and then use it as a stratifier for the following analyses.

For each drug, we derived a sample matched (with a 1:1 ratio) on the PS and compared the probability of being treated (i.e., PS itself) between females and males with *t* tests. Analyses were performed using the functions “matchit” and “match.data” from the MatchIt R package,^45^ using logistic regression to estimate the PS and the nearest neighbor method for matching the cohorts.

To further study statin exposure differences, we applied a logistic regression model and conditional inference tree (to visually illustrate associations between selected covariates and response) on the matched cohort (where the PS is computed based on treatment with statins). In both models we assess the exposure to statins on the basis of covariates not included in the PS analyses (i.e., sex, AD, dementia, and *APOE* ε4 genotype). Age, social‐economic status, education level, and relevant diagnoses for each drug were not included as covariates in the regression models as they were used to match the cohorts. We used the “rpart” and “rpart plot” function of the “rpart” package.^46^

After we assessed the probability of being treated with statins and identified key stratifiers, we evaluated the effect of statins on specific outcomes (i.e., survival, AD prevalence, and cognitive decline) in the matched cohort.

To examine the effect of statin use on survival, we used death records captured by the UK Biobank. We used baseline measurements to build a survival model, left‐censored at baseline. Right‐censoring was applied at the last follow‐up date or date of death (if occurred). Survival was studied with a Cox regression model adjusted by sex, *APOE* genotype, AD diagnosis, and dementia diagnosis. We performed the analysis with the “coxph” function of the “survival” package.^47^

For assessing longitudinal cognitive patterns in relation to statin use we included individuals who had at least two measurements including baseline assessment. This selection of participants may have introduced some bias but was essential to determining slope of change in cognitive measures.

To test for differences in the rate of change of the cognitive measures between statin‐user and non‐user groups over follow‐ups we used a linear mixed‐effects model (using the “lme4” package^48^) including the visit (time) effect, interaction terms with statins treated/non‐treated groups, and adjusted for sex and *APOE* genotype. To further study intra‐individual variability of RT over years we computed the slope of RT over time (Slope.yrs) as the difference of the measure at follow up and the baseline divided by the time in‐between the two measures (Equation 1). Higher Slope.yrs values indicate greater deterioration of cognitive function in time, while negative values indicate improvements.

(1) $$Slope.yrs\;=\frac{RT\left(fup\right)-RT\left(basline\right)}{Time.yrs\left(fup\right)-Time.yrs\left(baseline\right)}$$

To examine potential effects of statins use on AD prevalence, we conducted a cross‐sectional analysis on individuals who were diagnosed with AD at baseline and were *APOE* genotyped in the matched data set. Longitudinal information was not available for these participants, likely due to loss of follow‐up or dropout from the study. Therefore, we analyzed the prevalence of AD with a multivariate logistic regression model with statin use, *APOE* genotype, and sex as covariates as well as all two‐way interactions between these covariates (*APOE* × sex, *APOE* × statin use, sex × statin use).

## 3 RESULTS

### 3.1 The UK Biobank aging population

From the entire UK Biobank cohort, 252,327 who were aged 55 or over at recruitment (baseline) had a determined *APOE* genotype and baseline data, and were selected for our investigations (Table 1). Of these, 14,523 (4.717%) had data available from their first follow‐up visit and 2,677 (0.87%) had data available from baseline, first, and second follow‐up visits (for a full description of selection criteria for each analysis see Figure S1 in supporting information).

We found no differences in the population distribution in the four main classes (defined by sex and *APOE* genotypes), nor were there any differences when stratified by ethnicity.

A comparison of females (n = 136,665) and males (n = 115,662) revealed that the two groups differ in terms of age, education level, cognitive measures, disease diagnoses, and statin use, but not in Townsend deprivation or AD incidence.

Data illustrate that in the selected population, males are older, have a higher level of education, better cognitive scores, higher incidence of cardiovascular and metabolic diseases, and higher rate of statins use. We further compared males and females stratified by *APOE* genotypes (carriers vs. non‐carriers of the *APOE* ε4 allele). In both females and males, statistically significant differences were found for disease diagnoses, including AD and dementia, and in use of different statins, excluding pravastatin in males.

Dementia and AD diagnoses do not overlap for the majority of the cases, except in 150 subjects, which represents 10.8% of the total population with a diagnosis of dementia or AD (n = 1390).

As for cognitive measures at baseline, only RT was found statistically significant different in both females and males comparing *APOE* ε4 carriers and non‐carriers.

### 3.2 Drug exposure in the aging population

**FIGURE 2:**
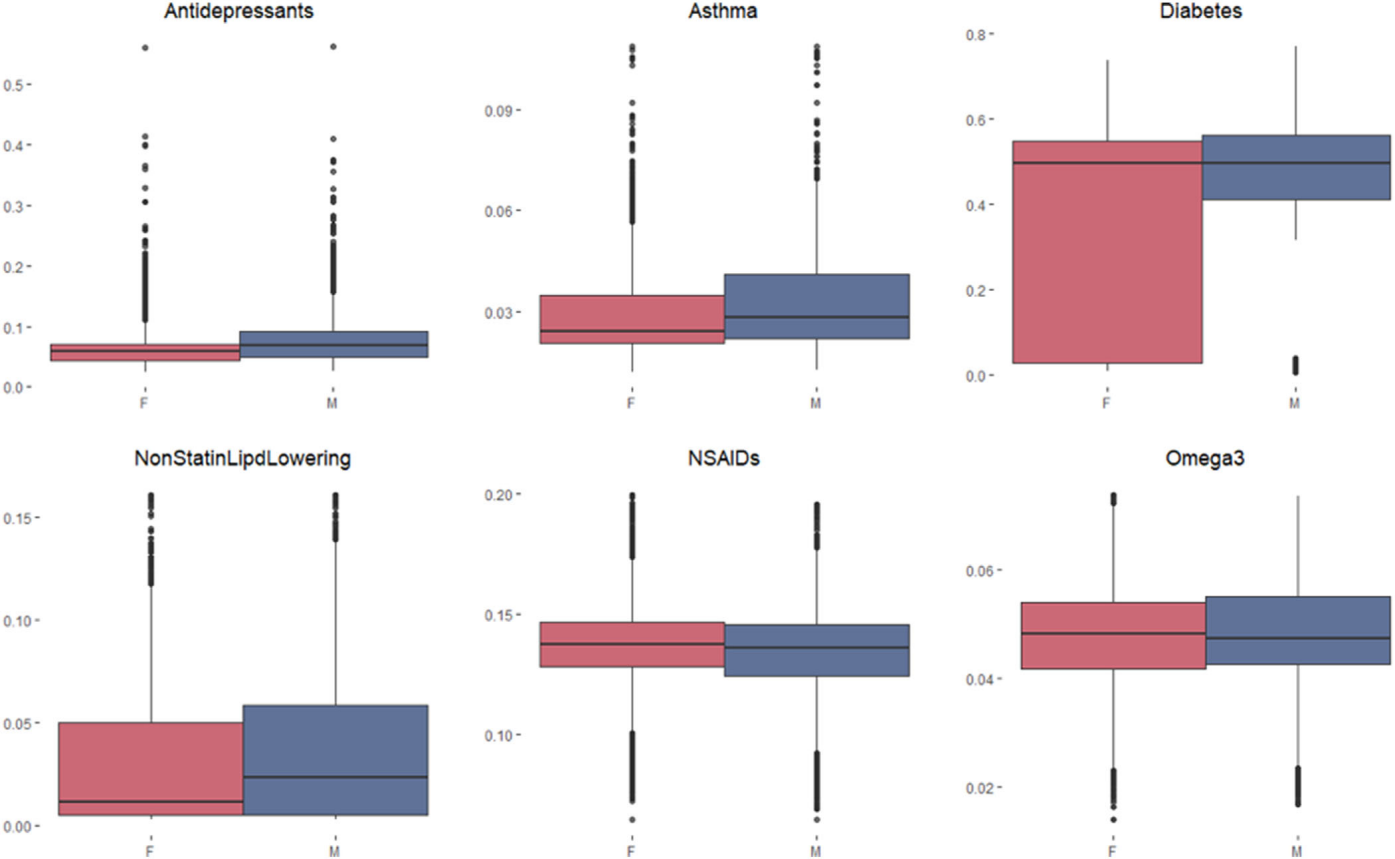
Drug exposure propensity scores in females (red) and males (blue) in the matched data sets

To assess drug exposure in the aging population, datasets matched via PS were created for each drug (Table 2). Further results regarding the matching process for statins treatments are reported in Appendix D in supporting information. We observed significant differences in drug exposure between females and males (Figure 2). Females are less likely to be treated with antidepressants, asthma medication, diabetes drugs, and non‐statin lipid lowering drugs; and more likely to be treated with NSAIDs and omega 3s.

**TABLE 2:** Comparison among female and male of propensity scores in each matched data set

Drug	Matched data (N)	PS in female mean (SD)	PS in male mean (SD)	*P*
AD medications	152	0.04(0.06)	0.04(0.06)	.57
Antidepressant	27578	0.06(0.02)	0.07(0.03)	< .001**
Asthma	13012	0.02(0.1)	0.03(0.1)	< .001**
Diabetes	24554	0.36(0.2)	0.40(0.2)	< .001**
Non statins lipid lowering	5164	0.02(0.03)	0.04(0.03)	< .001**
NSAIDs	66224	0.137(0.01)	0.135(0.1)	< .01*
Omega 3	22340	0.0479(0.009)	0.0475(0.009)	< .01*

PS values are compared via *t* test.

* *P*‐val < = .05.

** *P*‐val < = .01.

Abbreviations: AD, Alzheimer's disease; NSAID, non‐steroidal anti0inflammatory drug; PS, propensity score.

To examining statin exposure differences, we applied a logistic regression model and conditional inference tree to assess the exposure in the matched dataset (Table S1 in supporting information) on the basis of features not included in the PS analyses (i.e., sex, AD and dementia diagnoses, and *APOE* ε4 genotype—indicated in Table S1 as non‐matched).

Based on the regression model, males (*z*‐value = 51.2, odds ratio [OR] 1.84 [2.5% 1.8 to 7.5% 1.89], *P*‐value < .0001) and participants with an *APOE* ε4–positive genotype (*z*‐value = 10.6, OR 1.17 [2.5% 1.13 to 97.5% 1.2], *P*‐value < .0001), have a higher probability of being treated with statins. In this population, participants with an AD diagnosis were slightly less likely to be treated with statins (*z*‐value = –3.0, OR 0.64 [2.5% 0.48 to 97.5% 0.85], *P*‐value = .0025). Table S2 in supporting information reports model outputs.

**FIGURE 3:**
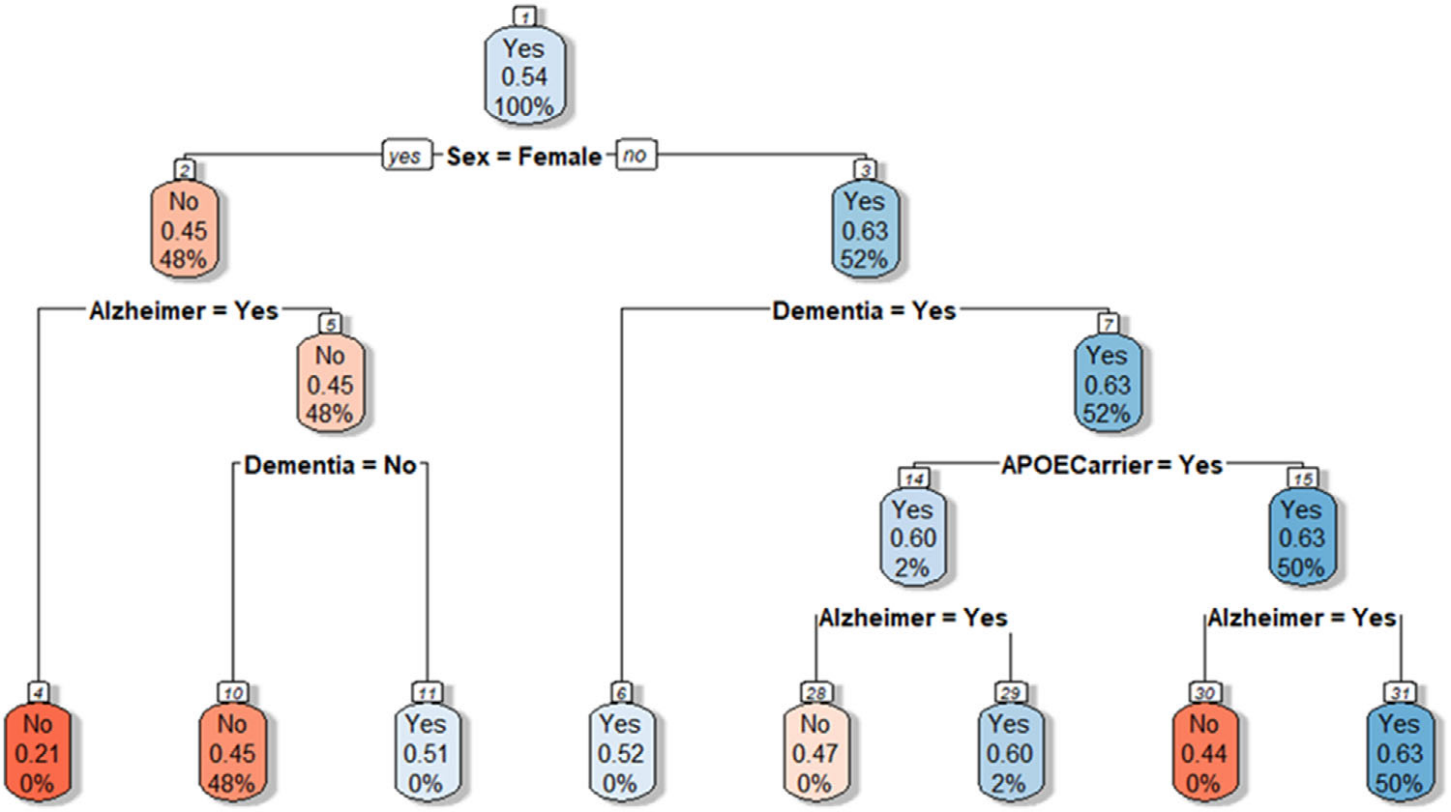
Results of the tree model. Each node shows the predicted class (Yes = treated or No = not treated). Color legend indicates the fitted value. Each tree node reports the predicted class, the predicted probability of the class (i.e., of being treated), and the actual percentage of observations in the node belonging to the class. Branches indicate the value of the variable for which the node was split. For example, the first node includes the whole population, split on the basis of sex; node two indicates the female population, where the probability of being treated is 0.45, the predicted class in “No”; node three indicates the male population, where the probability of being treated is 0.63, the predicted class in “Yes”

A second approach to visually illustrate statin exposure differences by stratifiers, is based on recursive partitioning and reports the results as logical tree structures (Figure 3). Treatment with statins is stratified by sex, *APOE* ε4 genotype, and degenerative diseases. However, the model suggests that treatment is stratified by *APOE* ε4 genotype in males (nodes 14 and 15), but not in females. Tree models also provide lists of rules, which summarize the branch path to each final node and its predicted probability. Within our model, the rule associated with the lowest probability of being treated (0.21) is the one including females without a diagnosis of AD (node 4); while the one with the highest probability of being treated (0.63) is that which includes males diagnosed with AD or dementia and who have an *APOE* ε4 genotype (node 31). For completeness, we further performed these analyses including all possible *APOE* genotypes (*APOE* ε2, *APOE* ε3, and *APOE* ε4) as possible stratifiers. The full results are provided in Figure S2 in supporting information.

### 3.3 Effects of exposure to statins

To examine the effect of statin use on survival, death records captured by the UK Biobank were used. We performed the following analysis on the dataset matched on statin PS, thus including as covariates sex, *APOE* genotype, AD, dementia diagnoses, and their interactions with statin treatment.

The matched data set included 6622 death events (3170 in statin users and 3452 in non‐users). The multivariate Cox regression analysis (Table 3 and Figure S4 in supporting information) revealed that use of statins was not associated with overall higher rates of survival (*P*‐value = .206). On the other hand, considering the interaction of statin use with sex, the results suggest higher survival rates in males treated with statins.

**TABLE 3:** Results from the Cox regression model of survival

	Estimate	Standard error	*Z*.value	*P*
Statin‐treated	0.92062	0.0654	‐1.265	.206
*APOE* ε4 carriers	1.14758	0.05871	2.345	.019*
Sex (male)	1.7755	0.06404	8.964	< .001**
AD diagnosis	2.34424	0.14309	5.954	< .001**
Dementia diagnosis	6.49512	0.07762	24.104	< .001**
*APOE* ε4 carriers: sex (male)	0.91895	0.0654	‐1.292	.1962
Sex (male): statin‐treated	1.1226	0.05223	2.214	.0268*
*APOE* ε4 carriers: statin‐treated	1.02295	0.06262	0.362	.7172

* *P*‐val < = .05.

** *P*‐val < = .001.

Abbreviations: AD, Alzheimer's disease; APOE, apolipoprotein E.

As suggested by our analyses, individuals differ in probability of statin use on the basis of strata defined by sex and *APOE* genotype. Here we examined whether differences in use of statins have an effect on RT changes.

To assess changes in cognitive patterns measured by RT, we included individuals who had at least two measurements (from two visits) after baseline assessment. The average length of time (days) between baseline and first follow‐up was 1,565.64 ± 343.2, and 962.66 ± 288.6 between first and second follow‐up visits. A total of 3877 individuals from the matched cohort had available RT measures (milliseconds) data at least two visits (Figure S3 in supporting information).

**FIGURE 4:**
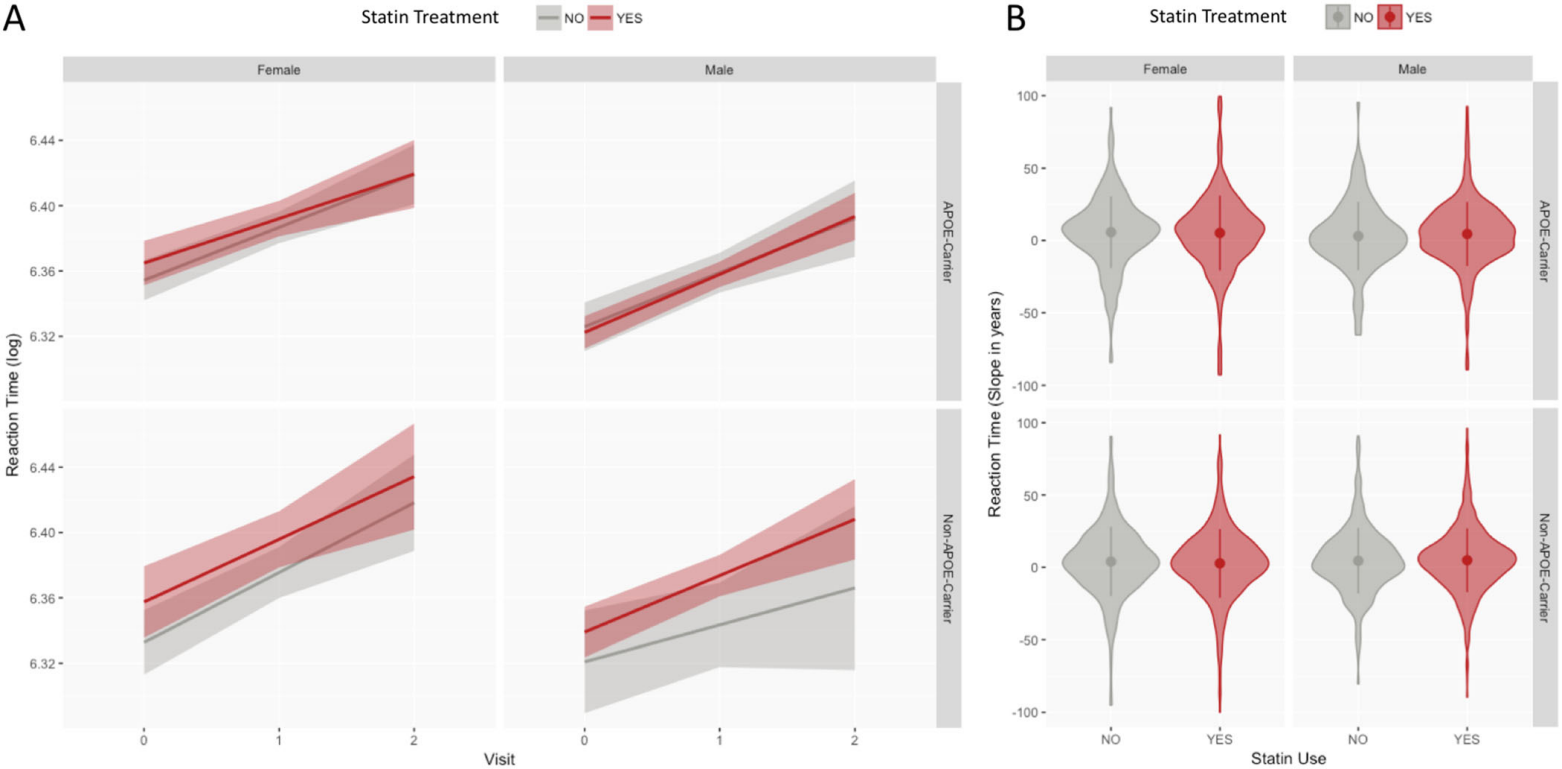
Comparison of statin users (red) and non‐users (gray) in the different population strata. For each of the four strata, the figure reports Reaction Time (RT) log‐transformed scores in time, and the RT Slope.yrs in the observation period

A linear mixed effects model was used to test differences in the RT rate of change over the entire follow‐up period (three time points) in the statins matched dataset. The model includes a random effect term indicating variation over time in each subject (Time from baseline|Subject), and adjusted for sex, *APOE* genotype, and their interactions with statins treatments (Table 4). Changes in RT were significantly associated with time from baseline (scores worsened in time, as already described in Lyall et al.^39^) as well as sex; males had worse performance over time. Significant differences (*P* = .03) were found in RT changes between statin users and non‐users when stratified by *APOE* genotype, as can be seen in Figure 4.

**TABLE 4:** Analysis of variance table from linear mixed effect model of the rate of change in reaction time measures between the statin‐users and non‐users

	Sum Sq	Mean Sq	NumDF	DenDF	F.value	Pr(> F)
Statin users (yes)	0.0058	0.0058	1	3864.1	0.382	0.53675
Time (follow‐up)	4.8107	4.8107	1	4463.5	315.551	<2.20E‐16**
*APOE* ε4 carriers	0.0007	0.0007	1	3870.9	0.045	0.83219
Sex (male)	0.5119	0.5119	1	3863.8	33.58	7.39E‐09**
Statins: *APOE* ε4 carriers	0.0695	0.0695	1	3870.6	4.556	0.03286*
Statins:sex	0.0058	0.0058	1	3864.1	0.382	0.53675

* *P*‐val < = .05.

** *P*‐val < = .001.

Abbreviations: APOE, apolipoprotein E.

Figure 4A illustrates RT scores at time points (0 = baseline, 1 = first visit, 2 = second visit) in statin users (red) and non‐users (gray) in the strata suggested by the model (male and female, and *APOE* ε4 carriers and non‐carriers). As suggested by the mixed effect model (Table 4), significant differences are observed only when the interaction between treatment and *APOE* ε4 genotype is considered. In general, statin users have worse RT score, but these differences are reduced in *APOE* ε4 carriers. Specifically, in male *APOE* ε4 carriers, statin users and non‐users demonstrate substantial overlap of RT scores in time and, while not statistically significant, they are the only strata in which RT is higher in non‐users (mean = 6.33, standard deviation [SD] = 0.1) than in user (mean = 6.32, SD = 0.1) at baseline.

We tested the differences in RT variations in time (Slope.yrs) between statin users and non‐users in strata (see Figure 4B). Larger slopes indicate faster cognitive deterioration. No significant differences were seen. However, as suggested by Figure 4B, different behaviors in deterioration can be seen: in females with an *APOE* ε4 carrier genotype, statin non‐users deteriorate faster (mean RT slope = 6.24 [mms/y]) than statin users (mean RT slope = 6.02 [mms/y]); this is unlike males without an *APOE* ε4 carrier genotype, in whom statin non‐users deteriorate slower (mean RT slope = 4.70 [mms/y]) than users (mean RT slope = 4.77 [mms/y]).

### 3.4 Statin use, dementia, and AD

To examine potential effects of statin on AD prevalence, a multivariate logistic regression model including statin use, *APOE* genotype, and sex as interaction terms found that *APOE* ε4 carriers demonstrate increased risk for AD (*z*‐value = 11.05, OR 4.88 [2.5% 3.68 to 97.5% 6.46], *P* < .0001). More interestingly, while statin users have increased risk of AD (*z*‐value = 3.76, OR 2.00 [2.5% 1.4 to 97.5% 2.88], *P* = .00017), *APOE* ε4 carriers, reported to be using statins, appear to have a decreased risk for AD (*z*‐value = –1.77, OR 0.69 [2.5% 0.46 to 97.5% 1.04], *P* = .07), though non‐significant in our analysis. Table S3 in supporting information reports the full model output.

We further carried out this analysis to investigate the effect of statins on the prevalence of dementia. Here, we found that *APOE* ε4 carriers also have an increased risk for dementia (*z*‐value = 11.05, OR 3.15 [2.5% 2.53 to 97.5% 3.92], *P* < .0001). Further, while again statin‐using *APOE* ε4 carriers showed a non‐significant decreased risk for dementia (*z*‐value = –1.74, OR 0.78 [2.5% 0.58 to 97.5% 1.03], *P* = .08), males showed an increased risk for dementia (*z*‐value = 6.34, OR 1.84 [2.5% 1.52 to 97.5% 2.22], *P* < .0001), while males with an *APOE* ε4 genotype show a significant reduced risk for dementia (*z*‐value = –2.952, OR 0.66 [2.5% 0.5 to 97.5% 0.87], *P* = .003). The full results are reported in Table S4 in supporting information.

## 4 DISCUSSION

Statins have greater beneficial effects on cognitive function in *APOE* ε4 homozygotes,^11^ and it has been demonstrated^35^ that a reduction in AD risk is associated with statin use and varies across sex and ethnicity. The UK Biobank allowed us to further examine potential effects of statin in the aging and AD populations, stratified by sex and *APOE* genotype.

Our results support sex differences related to statin use in the aging population; this in accordance with other studies.^49^, ^50^, ^51^, ^52^ We found that participants with an AD diagnosis were slightly less likely to be treated with statins; this may be due to reverse causation, where statins are more likely prescribed to patients who are not cognitively impaired and would adhere to treatment. Most strikingly, when we examined statin exposure differences while allowing for multilevel stratification, we found significant differences in the strata which contribute to the probability of statin use. *APOE* genotype is correlated to differences in rates of treatment with statins in males but not in females. Multivariate survival analysis revealed that changes in survival are associated to the use of statins only when accounting for the interaction with sex strata. Past studies have shown that women are less likely to be treated with statin therapy,^49^, ^50^ and as *APOE* ε4 carriers have increased risk for dyslipidemia; this likely increases the likelihood of patients with this genotype to be treated with statins.^53^

While the associations between statin treatment and AD have been explored in the past,^23^, ^35^ this study takes a step further to examine the interaction of sex and *APOE* with cognitive decline. Our results indicate that statins may have a beneficial effect on cognitive functions, however limited to specific combinations of sex strata and *APOE* ε4 genotypes. Analysis of cognitive measures in statin users versus non‐users suggests that males with an *APOE* ε4 genotype may benefit more from use of statins; however, this analysis was somewhat limited due to the size of available subcohorts.^49^, ^50^ Further, and in accordance with previous findings,^11^ we found preliminary indications (though not statistically significant) that *APOE* ε4 carriers, reported to be using statins, might be associated with a decreased risk of AD.

One significant limitation is that cognitive measures available in the UK Biobank may not fully capture cognitive changes over time in the non–clinically impaired population. While RT has been successfully used to assess cognitive impairment in aging and dementia populations,^54^, ^55^, ^56^ there is little evidence in the literature for its possible use in the context of AD and its use as a preclinical AD marker.^57^ Finer changes may be better captured with more robust, specific, diagnostically designed measures such as the Mini‐Mental State Examination^58^ or Alzheimer's Disease Assessment Scale–cognitive subscale (ADAS‐cog).^59^ We could not conduct longitudinal analysis of change in cognition in the AD cohort as data were not available. This is most likely due to dropout, as individuals diagnosed with AD are less likely to follow‐up with a study such as the UK Biobank. Nevertheless, our analyses revealed that statin use in *APOE* ε4 carriers decreases the risk for AD, in alignment with findings from previous studies.^11^

Another valuable addition to the analyses presented here would be a further stratification of the patients according to biochemical markers such as cholesterol or triglyceride levels in plasma. Future analyses should include this information.

The UK Biobank has several potential biases: general ones such as the enrollment of a mostly White population, with higher socioeconomic status, and specific for study, including possible selection biases, such as higher rates of depression in females. While the UK Biobank's cohort contains a mixture of prevalent conditions, including dementia and AD, it is important to note that ICD‐10‐CM codes might not always be accurate, particularly for these types of diagnoses. Furthermore, given the observational nature of the UK Biobank data, our results showing that statin use was associated with lower risk of cognitive impairment in AD among *APOE* ε4 carriers, needs to be further validated by a randomized clinical trial.

Our evaluation of the aging population in a large‐scale cohort from the UK Biobank identified important sex differences related to statin use. Our results suggest that patient stratification that includes *APOE* genotype and consciousness of sex bias could significantly reduce risk of AD in both men and women.

## CONFLICTS OF INTEREST

The authors have no financial conflicts of interest.

## ETHICAL APPROVAL AND CONSENT TO PARTICIPATE

This research has been conducted using the UK Biobank Resource under Application Number 19923 “A precision medicine approach for treatment and prevention of Alzheimer's disease using statins.” UK Biobank has approval from the North West Multi‐centre Research Ethics Committee (MREC), which covers the UK. All participants have previously provided consent for UK Biobank data and samples to be used for research.

## AVAILABILITY OF DATA AND MATERIALS

The data that support the findings of this study are available from UK Biobank. Restrictions apply to the availability of these data, which were used under the application 19923 license for the current study, and so are not publicly available.

## AUTHOR CONTRIBUTIONS

Arianna Dagliati performed experiments, analyzed data, and wrote the manuscript draft. Arianna Dagliati, Nophar Geifman, Niels Peek, and Roberta Diaz Brinton designed and developed the study. All authors revised and edited the manuscript.

## Supporting information

Supporting information.

## References

[1] Beard JR , Officer AM , Cassels AK . The world report on ageing and health. Gerontologist. 2016;56(Suppl 2):S163‐S166. 26994257 10.1093/geront/gnw037

[2] Eurostat . People in the EU: who are we and how do we live? 2015. Office of the European Union. 2015. https://ec.europa.eu/eurostat/web/products-statistical-books/-/KS-04-15-567

[3] Government office for science . Future of an ageing population. 2016. https://www.gov.uk/government/publications/future‐of‐an‐ageing‐population.

[4] Boards of trustees of the Federal Hospital Insurance and Federal Supplementary Medical Insurance Trust Funds . 2019 Annual report of the boards of trustees of the Federal Hospital Insurance and Federal Supplementary Medical Insurance Trust Funds. 2019. https://www.cms.gov/Research‐Statistics‐Data‐and‐Systems/Statistics‐Trends‐and‐Reports/ReportsTrustFunds/Downloads/TR2019.pdf.

[5] Seifarth JE , McGowan CL , Milne KJ . Sex and life expectancy. Gend Med. 2012;9(6):390‐401. 23164528 10.1016/j.genm.2012.10.001

[6] Regan JC , Partridge L . Gender and longevity: why do men die earlier than women? Comparative and experimental evidence. Best Pract Res Clin Endocrinol Metabol. 2013;27(4):467‐749. 10.1016/j.beem.2013.05.016 24054925

[7] Barrett ELB , Richardson DS . Sex differences in telomeres and lifespan. Aging Cell. 2011;10(6):913‐321. 21902801 10.1111/j.1474-9726.2011.00741.x

[8] Brundtland GH . Men Ageing and Health: Achieving Health Across the Life Span. WHO; 2001.

[9] Hersi M , Irvine B , Gupta P , Gomes J , Birkett N , Krewski D . Risk factors associated with the onset and progression of Alzheimer's disease: a systematic review of the evidence. Neurotoxicology. 2017;61:143‐187. 28363508 10.1016/j.neuro.2017.03.006

[10] Martins IJ , Berger T , Sharman MJ , Verdile G , Fuller SJ , Martins RN . Cholesterol metabolism and transport in the pathogenesis of Alzheimer's disease. J Neurochem. 2009;111(6):1275‐1308. 20050287 10.1111/j.1471-4159.2009.06408.x

[11] Geifman N , Brinton RD , Kennedy RE , Schneider LS , Butte AJ . Evidence for benefit of statins to modify cognitive decline and risk in Alzheimer's disease. Alzheimer's Res Ther. 2017;9(1):10. 28212683 10.1186/s13195-017-0237-y PMC5316146

[12] Haag MDM , Hofman A , Koudstaal PJ , Stricker BHC , Breteler MMB . Statins are associated with a reduced risk of Alzheimer disease regardless of lipophilicity. The Rotterdam Study. J Neurol Neurosurg Psychiatry. 2009;80(1):13‐17. 18931004 10.1136/jnnp.2008.150433

[13] Jick H , Zornberg GL , Jick SS , Seshadri S , Drachman DA . Statins and the risk of dementia. Lancet. 2000;356(9242):1627‐1631. 11089820 10.1016/s0140-6736(00)03155-x

[14] Rockwood K , Kirkland S , Hogan DB , et al. Use of lipid‐lowering agents, indication bias, and the risk of dementia in community‐dwelling elderly people. Arch Neurol. 2002;59(2):223‐227. 11843693 10.1001/archneur.59.2.223

[15] Wolozin B , Kellman W , Ruosseau P , Celesia GG , Siegel G . Decreased prevalence of Alzheimer disease associated with 3‐hydroxy‐3‐methyglutaryl coenzyme a reductase inhibitors. Arch Neurol. 2000;57(10):1439‐1443. 11030795 10.1001/archneur.57.10.1439

[16] Sparks DL , Kryscio RJ , Sabbagh MN , Connor DJ , Sparks LM , Liebsack C . Reduced risk of incident AD with elective statin use in a clinical trial cohort. Curr Alzheimer Res. 2008;5(4):416‐421. 18690839 10.2174/156720508785132316

[17] Dergunov AD . Apolipoprotein E genotype as a most significant predictor of lipid response at lipid‐lowering therapy: mechanistic and clinical studies. Biomed Pharmacother. 2011;65(8):597‐603. 21705182 10.1016/j.biopha.2011.04.003

[18] Feldman HH , Doody RS , Kivipelto M , et al. Randomized controlled trial of atorvastatin in mild to moderate Alzheimer disease. Neurology. 2010;74(C):956‐964. 20200346 10.1212/WNL.0b013e3181d6476a

[19] Mcguinness B , Craig D , Bullock R , Malouf R , Passmore P . Statins for the treatment of dementia. Cochrane Database of Systematic Reviews. 2014(7):CD007514. 10.1002/14651858.CD007514.pub3 PMC11112650 25004278

[20] Sano M , Bell K , Galasko D , et al. A randomized, double‐blind, placebo‐controlled trial of simvastatin to treat Alzheimer disease. Neurology. 2011;77:556‐563. 21795660 10.1212/WNL.0b013e318228bf11 PMC3149154

[21] Simons M , Schwärzler F , Lütjohann D , et al. Treatment with simvastatin in normocholesterolemic patients with Alzheimer's disease: a 26‐week randomized, placebo‐controlled, double‐blind trial. Ann Neurol. 2002;52(3):346‐350. 12205648 10.1002/ana.10292

[22] Zissimopoulos JM , Barthold D , Brinton RD , Joyce G . Sex and race differences in the association between statin use and the incidence of Alzheimer disease. JAMA Neurol. 2017;74(2):225‐232. 27942728 10.1001/jamaneurol.2016.3783 PMC5646357

[23] Torrandell‐Haro G , Branigan GL , Vitali F , Geifman N , Zissimopoulos JM , Brinton RD . Statin therapy and risk of Alzheimer's and age‐related neurodegenerative diseases. Alzheimer's Dement. 2020;6(1):e12108. 10.1002/trc2.12108 PMC7687291 33283039

[24] Guan ZW , Wu KR , Li R , et al. Pharmacogenetics of statins treatment: efficacy and safety. J Clin Pharm The. 2019;44(6):858‐867. 10.1111/jcpt.13025 31436349

[25] Zhang L , He S , Li Z , et al. Apolipoprotein e polymorphisms contribute to statin response in Chinese ASCVD patients with dyslipidemia. Lipids Health Dis. 2019;18(1):129. 31153375 10.1186/s12944-019-1069-5 PMC6545221

[26] Baptista R , Rebelo M , Decq‐Mota J , et al. Apolipoprotein e epsilon‐4 polymorphism is associated with younger age at referral to a lipidology clinic and a poorer response to lipid‐lowering therapy. Lipids Health Dis. 2011;10:48. 21450082 10.1186/1476-511X-10-48 PMC3078893

[27] Banach M , Rizzo M , Nikolic D , Howard G , Howard VJ , Mikhailidis DP . Intensive LDL‐cholesterol lowering therapy and neurocognitive function. Pharmacol Ther. 2017;170:181‐191. 27865998 10.1016/j.pharmthera.2016.11.001

[28] Maitland‐Van Der Zee AH , Jukema JW , Zwinderman AH , et al. Apolipoprotein‐E polymorphism and response to pravastatin in men with coronary artery disease (REGRESS). Acta Cardiol. 2006;61(3):327‐331. 16869455 10.2143/AC.61.3.2014836

[29] Bennet AM , Di Angelantonio E , Ye Z , et al. Association of apolipoprotein e genotypes with lipid levels and coronary risk. JAMA. 2007;298(11):1300‐1311. 17878422 10.1001/jama.298.11.1300

[30] Nieminen T , Kähönen M , Viiri LE , Grönroos P , Lehtimäki T . Pharmacogenetics of apolipoprotein E gene during lipid‐lowering therapy: lipid levels and prevention of coronary heart disease. Pharmacogenomics. 2008;9(10):1475‐1486. 18855536 10.2217/14622416.9.10.1475

[31] de Oliveira FF , Chen ES , Smith MC , Bertolucci PHF . Selected LDLR and APOE polymorphisms affect cognitive and functional response to lipophilic statins in Alzheimer's Disease. J Mol Neurosci. 2020;70(10):1574‐1588. 32474901 10.1007/s12031-020-01588-7

[32] Gutierrez J , Ramirez G , Rundek T , Sacco RL . Statin therapy in the prevention of recurrent cardiovascular events a sex‐based meta‐analysis. Arch Intern Med. 2012;172(12):909‐919. 22732744 10.1001/archinternmed.2012.2145

[33] Pedro‐Botet J , Schaefer EJ , Bakker‐Arkema RG , et al. Apolipoprotein E genotype affects plasma lipid response to atorvastatin in a gender specific manner. Atherosclerosis. 2001;158(1):183‐193. 11500190 10.1016/s0021-9150(01)00410-5

[34] Puri R , Nissen SE , Shao M , et al. Sex‐related differences of coronary atherosclerosis regression following maximally intensive statin therapy: insights from saturn. JACC Cardiovasc Imaging. 2014;7(10):1013‐1022. 25240453 10.1016/j.jcmg.2014.04.019

[35] Zissimopoulos JM , Barthold D , Brinton RD , Joyce G . Sex and race differences in the association between statin use and the incidence of Alzheimer disease. JAMA Neurol. 2017;74(2):225‐232. 27942728 10.1001/jamaneurol.2016.3783 PMC5646357

[36] Sudlow C , Gallacher J , Allen N , et al. UK biobank: an open access resource for identifying the causes of a wide range of complex diseases of middle and old age. Plos Med. 2015;12(3):e1001779. 25826379 10.1371/journal.pmed.1001779 PMC4380465

[37] Ferket BS , Hunink MGM , Khanji M , Agarwal I , Fleischmann KE , Petersen SE . Cost‐effectiveness of the polypill versus risk assessment for prevention of cardiovascular disease. Heart. 2017;103(7):483‐491. 28077465 10.1136/heartjnl-2016-310529

[38] Davies G , Marioni RE , Liewald DC , et al. Genome‐wide association study of cognitive functions and educational attainment in UK Biobank (N = 112 151). Mol Psychiatry. 2016;21(August 2015):1‐10. 27046643 10.1038/mp.2016.45 PMC4879186

[39] Lyall DM , Cullen B , Allerhand M , et al. Cognitive test scores in UK biobank: data reduction in 480,416 participants and longitudinal stability in 20,346 participants. PLoS One. 2016;11(4)):e0154222. 27110937 10.1371/journal.pone.0154222 PMC4844168

[40] Hendrie HC , Hake A , Lane K , et al. Statin use, incident dementia and Alzheimer disease in elderly African Americans. Ethn Dis. 2015;25(3):345‐354. 26673814 10.18865/ed.25.3.345 PMC4671419

[41] Piumatti G , Moore SC , Berridge D , Sarkar C , Gallacher J . The relationship between alcohol use and long‐term cognitive decline in middle and late life: a longitudinal analysis using UK Biobank. J Public Health. 2018;40(2):1‐8. 10.1093/pubmed/fdy032 29462350

[42] National Research Council , 2011. Toward precision medicine: building a knowledge network for biomedical research and a new taxonomy of disease. https://pubmed.ncbi.nlm.nih.gov/22536618/ 22536618

[43] Bycroft C , Freeman C , Petkova D , et al. The UK Biobank resource with deep phenotyping and genomic data. Nature. 2018;562(7726):203‐209. 30305743 10.1038/s41586-018-0579-z PMC6786975

[44] Bycroft C , Freeman C , Petkova D , et al. Genome‐wide genetic data on ∼500,000 UK Biobank participants. BioRxiv. 2017; 166298.

[45] Ho DE , Imai K , King G , Stuart EA . MatchIt: nonparametric preprocessing for parametric causal inference. J Stat Softw. 2011;42(8):1‐28.

[46] Hothorn T , Zeileis A . Partykit: a modular toolkit for recursive partytioning in R. J Mach Learn Res. 2015;16(1):3905–3909.

[47] Therneau TM , Lumley T . Package ‘survival.’. R Top Doc. 2015;128(10):28–33.

[48] Bates D , Mächler M , Bolker BM , Walker SC . Fitting linear mixed‐effects models using lme4. J Stat Softw. 2015; 10.18637/jss.v067.i01

[49] Karalis DG , Wild RA , Maki KC , et al. Gender differences in side effects and attitudes regarding statin use in the Understanding Statin Use in America and Gaps in Patient Education (USAGE) study. J Clin Lipidol. 2016;10(4):833‐841. 27578114 10.1016/j.jacl.2016.02.016

[50] Nanna MG , Wang TY , Xiang Q , et al. Sex differences in the use of statins in community practice. Circ Cardiovasc Qual Outcomes. 2019;12(8):e005562. 31416347 10.1161/CIRCOUTCOMES.118.005562 PMC6903404

[51] Peters SAE , Colantonio LD , Zhao H , et al. Sex differences in high‐intensity statin use following myocardial infarction in the United States. J Am Coll Cardiol. 2018;71(16):1729‐1737. 29673463 10.1016/j.jacc.2018.02.032

[52] Zhao M , Woodward M , Vaartjes I , et al. Sex differences in cardiovascular medication prescription in primary care: a systematic review and meta‐analysis. J Am Heart Assoc. 2020;9(11):e014742. 32431190 10.1161/JAHA.119.014742 PMC7429003

[53] Mahley RW , Rall SC . Apolipoprotein E: far more than a lipid transport protein. Annu Rev Genomics Hum Genet. 2000;1:507‐537. 11701639 10.1146/annurev.genom.1.1.507

[54] Chen KC , Weng CY , Hsiao S , Tsao WL , Koo M . Cognitive decline and slower reaction time in elderly individuals with mild cognitive impairment. Psychogeriatrics. 2017;17(6):364‐370. 28261945 10.1111/psyg.12247

[55] Kochan NA , Bunce D , Pont S , Crawford JD , Brodaty H , Sachdev PS . Reaction time measures predict incident dementia in community‐living older adults: the Sydney memory and ageing study. Am J Geriatr Psychiatry. 2016;24(3):221‐231. 26905045 10.1016/j.jagp.2015.12.005

[56] Phillips M , Rogers P , Haworth J , Bayer A , Tales A . Intra‐individual reaction time variability in mild cognitive impairment and Alzheimer's disease: gender, processing load and speed factors. PLoS One. 2013;8(6):e65712. 23762413 10.1371/journal.pone.0065712 PMC3677873

[57] Gorus E , De Raedt R , Lambert M , Lemper JC , Mets T . Reaction times and performance variability in normal aging, mild cognitive impairment, and Alzheimer's disease. J Geriatr Psychiatry Neurol. 2008;21(3):204‐218. 18838743 10.1177/0891988708320973

[58] Folstein MF , Folstein SE , McHugh PR . Mini‐mental state. A practical method for grading the cognitive state of patients for the clinician. J Psychiatr Res. 1975;12(3):189‐198. 1202204 10.1016/0022-3956(75)90026-6

[59] Rosen WG , Mohs RC , Davis KL . A new rating scale for Alzheimer's disease. Am J Psychiatry. 1984;141(11):1356‐1364. 6496779 10.1176/ajp.141.11.1356

