# Supplementary material for: Sex and APOE genotype differences related to statin use in the aging population
Source: Alzheimers Dement (N Y). 2021 May 2;7(1):e12156. doi: 10.1002/trc2.12156 (PMC8088592; doi:10.1002/trc2.12156)

# Appendix A – Drugs Codes Set

| **Type** | **Name** | **CODING_UKBiobank** |
| --- | --- | --- |
| ADRx (AD treatments) | Hydergine | 1140861424 |
|  | donepezil | 1141150834 |
|  | aricept | 1141150840 |
|  | rivastigmine | 1141167690 |
|  | Exelon | 1141167700 |
|  | Reminyl | 1141171482 |
|  | galantamine | 1141171578 |
|  | memantine | 1141182732 |
| Antidepressants | Buspar | 1140863454 |
|  | Prothiaden | 1140867624 |
|  | Doxepin | 1140867640 |
|  | Elavil | 1140867658 |
|  | Anafranil | 1140867690 |
|  | Tofranil | 1140867712 |
|  | Lofepramine | 1140867726 |
|  | Trimipramine | 1140867756 |
|  | Surmontil | 1140867758 |
|  | Vivalan | 1140867770 |
|  | Amoxapine | 1140867774 |
|  | Ludiomil | 1140867784 |
|  | Bolvidon | 1140867806 |
|  | Norval | 1140867812 |
|  | Nortriptyline | 1140867818 |
|  | Phenelzine | 1140867850 |
|  | Nardil | 1140867852 |
|  | Isocarboxazid | 1140867856 |
|  | Marplan | 1140867858 |
|  | Prozac | 1140867876 |
|  | Sertraline | 1140867878 |
|  | Paroxetine | 1140867888 |
|  | Tranylcypromine | 1140867914 |
|  | Parnate | 1140867916 |
|  | Moclobemide | 1140867920 |
|  | Manerix | 1140867922 |
|  | Amitriptyline | 1140867948 |
|  | Fluoxetine | 1140879540 |
|  | Fluvoxamine | 1140879544 |
|  | Maprotiline | 1140879552 |
|  | Mianserin | 1140879556 |
|  | Clomipramine | 1140879620 |
|  | Desipramine | 1140879624 |
|  | Imipramine | 1140879630 |
|  | Protriptyline | 1140879632 |
|  | Trazodone | 1140879634 |
|  | Selegiline | 1140879668 |
|  | Viloxazine | 1140879688 |
|  | Buspirone | 1140879730 |
|  | Buspirone | 1140879730 |
|  | Gamanil | 1140882310 |
|  | Sinequan | 1140882312 |
|  | Dosulepin | 1140909806 |
|  | Venlafaxine | 1140916282 |
|  | Nefazodone | 1140917460 |
|  | Citalopram | 1140921600 |
|  | Lomont | 1141146062 |
|  | Reboxetine | 1141151978 |
|  | Edronax | 1141151982 |
|  | Mirtazapine | 1141152732 |
|  | Thaden | 1141171824 |
|  | Bupropion | 1141176854 |
|  | Zyban | 1141176858 |
|  | Escitalopram | 1141180212 |
|  | Cipralex | 1141190158 |
|  | Atomoxetine | 1141199446 |
|  | Strattera | 1141199460 |
|  | Duloxetine | 1141200564 |
|  | Cymbalta | 1141201834 |
| Asthma treatments | Salmeterol | 1140862144 |
|  | Budesonide | 1140862572 |
|  | Pulmicort | 1140862574 |
|  | Flunisolide | 1140876154 |
|  | Beclomethasone | 1140881938 |
|  | Fluticasone | 1140888098 |
|  | Mometasone | 1140888172 |
|  | Montelukast | 1141157126 |
|  | Singulair | 1141157132 |
|  | QVAR | 1141167594 |
|  | Zafirlukast | 1141168340 |
|  | Accolate | 1141168344 |
|  | Symbicort | 1141174520 |
|  | Asmanex | 1141191818 |
|  | formoterol | 1141195224 |
| Diabetes treatments | acarbose | 1140868902 |
|  | glipizide | 1140874646 |
|  | tolazamide | 1140874664 |
|  | chlorpropamide | 1140874706 |
|  | Diabinese | 1140874712 |
|  | gliclazide | 1140874744 |
|  | Insulin | 1140883066 |
|  | metformin | 1140884600 |
|  | glimepiride | 1141152590 |
|  | Amaryl | 1141156984 |
|  | repaglinide | 1141168660 |
|  | pioglitazone | 1141171646 |
|  | Actos | 1141171652 |
|  | Starlix | 1141173786 |
|  | nateglinide | 1141173882 |
|  | rosiglitazone | 1141177600 |
|  | Avandia | 1141177606 |
|  | Avandamet | 1141189094 |
| Estrogens | Premarin | 1140868408 |
|  | Vagifem | 1140868472 |
|  | estropipate | 1140909906 |
|  | Estring | 1140911708 |
|  | estraderm | 1140926592 |
|  | estradiol | 1141181700 |
| Non-statin lipd-lowering | colestid | 1140861848 |
|  | gemfibrozil | 1140861856 |
|  | lopid | 1140861858 |
|  | questran | 1140861936 |
|  | fenofibrate | 1140861954 |
|  | colestipol | 1140888590 |
|  | niacin | 1140910670 |
|  | niaspan | 1141188546 |
|  | ezetimibe | 1141192736 |
| NSAIDs | Tolectin | 1140853030 |
|  | aspirin | 1140861806 |
|  | misoprostol | 1140865628 |
|  | salsalate | 1140871092 |
|  | etodolac | 1140871188 |
|  | Lodine | 1140871196 |
|  | fenoprofen | 1140871226 |
|  | flurbiprofen | 1140871236 |
|  | Arthrotec | 1140871266 |
|  | diflunisal | 1140871282 |
|  | Dolobid | 1140871284 |
|  | ibuprofen | 1140871310 |
|  | indomethacin | 1140871336 |
|  | naproxen | 1140871462 |
|  | Naprosyn | 1140871472 |
|  | ketoprofen | 1140871506 |
|  | Orudis | 1140871516 |
|  | Oruvail | 1140871522 |
|  | mefenamic | 1140871542 |
|  | sulindac | 1140871604 |
|  | Clinoril | 1140871606 |
|  | piroxicam | 1140871666 |
|  | Feldene | 1140871672 |
|  | tolmetin | 1140875268 |
|  | nabumetone | 1140875336 |
|  | Voltaren | 1140877872 |
|  | diclofenac | 1140878036 |
|  | Toradol | 1140881118 |
|  | ketorolac | 1140884558 |
|  | meloxicam | 1140926732 |
|  | Mobic | 1140926792 |
|  | Advil | 1140928656 |
|  | celecoxib | 1141176662 |
|  | celebrex | 1141176668 |
|  | etoricoxib | 1141180140 |
| Omega-3 | fishoil | 1193 |
|  | omega3 | 1193 |
|  | omega-3 | 1193 |
| Statins | simvastatin | 1140861958 |
|  | Lescol | 1140864592 |
|  | Zocor | 1140881748 |
|  | fluvastatin | 1140888594 |
|  | pravastatin | 1140888648 |
|  | Lipitor | 1141146138 |
|  | atorvastatin | 1141146234 |
|  | rosuvastatin | 1141192410 |
|  | Crestor | 1141192414 |
| VitaminE | AlphaE | 1140871112 |
|  | Aqua-E | 1140871112 |
|  | AquasolE | 1140871112 |
|  | Aquavite-E | 1140871112 |
|  | EPherol | 1140871112 |
|  | E-400 | 1140871112 |
|  | E-600 | 1140871112 |
|  | E-gems | 1140871112 |
|  | Nutr-E-Sol | 1140871112 |
|  | Vita-PlusE | 1140871112 |
|  | vitamine | 1140871112 |

# Appendix B – Additional analyses results.

**Table1.** Population characteristics in datasets matched on the basis of the statins PS (treated and not treated).

| Statins | Non-Treated | Treated |
| --- | --- | --- |
| N.pts | 57509 | 57509 |
| MATCHED FEATURES | | |
| Age At Recruitment | 63.59(3.8) | 63.43(3.9) |
| Age Completed Education | 16(3.5) | 15.94(3.5) |
| Townsend deprivation index | -1.2(3.2) | -1.03(3.2) |
|  |  |  |
| AMI | 2325(4%) | 4521(7%) |
| Atrial Fibrillation | 4954(8%) | 6227(10%) |
| Hypertension | 29953(52%) | 30221(52%) |
| Diabetes | 6154(10%) | 11507(20%) |
| Acute Cerebrovascular Disease | 993(1%) | 1300(2%) |
| Coronary Atherosclerosis | 7927(13%) | 14970(26%) |
| Disorders Lipid Metabolism | 10570(18%) | 20998(36%) |
| Angina | 5368(9%) | 11392(19%) |
|  |  |  |
| UN- MATCHED FEATURES | | |
| Sex(Male) | 24962(43%) | 33667(58%) |
| ApoE Carriers | 11375 (19%) | 12746 (22%) |
|  |  |  |
| Alzheimer | 117(0.2%) | 83(0.1%) |
| Dementia | 226(0.4%) | 281(0.5%) |

**Table2.** Logistic regression models to examine statins exposure differences.

|  | Estimate | Std.Error | z | Pr(>\|z\|) | Odds Ratio | 2.50% | 97.50% |
| --- | --- | --- | --- | --- | --- | --- | --- |
| (Intercept) | -0.344567 | 0.009108 | -37.831 | <2.00E-16 | 0.7085274 | 0.6959913 | 0.7212893 |
| Sex(M) | 0.611842 | 0.011945 | 51.22 | <2.00E-17 | 1.8438248 | 1.801158 | 1.8875024 |
| APOE | 0.155962 | 0.014692 | 10.615 | <2.00E-18 | 1.1687823 | 1.1356052 | 1.2029287 |
| Alzheimer | -0.448613 | 0.148145 | -3.028 | 0.00246 | 0.6385134 | 0.4776047 | 0.8536336 |
| Dementia | 0.156068 | 0.092341 | 1.69 | 0.091 | 1.1689057 | 0.9753912 | 1.4008129 |

**Table3.** Logistic regression models to examine AD prevalence.

|  | Estimate | Std.Error | z | Pr(>\|z\|) | Odds Ratio | 2.50% | 97.50% |
| --- | --- | --- | --- | --- | --- | --- | --- |
| (Intercept) | -7.37407 | 0.10682 | -69.032 | < 2.00E-16 | 0.000627307 | 0.00051 | 0.00077 |
| Sex(M) | 0.16992 | 0.15016 | 1.132 | 0.25779 | 1.18521144 | 0.88304 | 1.59078 |
| APOE | 1.58523 | 0.14342 | 11.053 | < 2.00E-16 | 4.8804325 | 3.68452 | 6.46451 |
| Statins | 0.69667 | 0.18532 | 3.759 | 0.00017 | 2.007062969 | 1.39577 | 2.88609 |
| APOE:Statins | -0.36618 | 0.20696 | -1.769 | 0.07683 | 0.69337765 | 0.46218 | 1.04024 |
| Sex(M):Statins | -0.23317 | 0.2076 | -1.123 | 0.26136 | 0.792017885 | 0.52726 | 1.18972 |
| Sex(M):APOE | -0.05308 | 0.19151 | -0.277 | 0.78166 | 0.948304465 | 0.65152 | 1.38027 |

**Table4.** Logistic regression models to examine Dementia prevalence.

|  | Estimate | Std.Error | z | Pr(>\|z\|) | Odds Ratio | 2.50% | 97.50% |
| --- | --- | --- | --- | --- | --- | --- | --- |
| (Intercept) | -6.67951 | 0.07557 | -88.385 | < 2e-16 | 0.001256394 | 0.001083423 | 0.001456979 |
| Sex(M) | 0.60875 | 0.09743 | 6.24 | 4.15e-10 | 1.838129671 | 1.518610847 | 2.224875909 |
| APOE | 1.58523 | 0.14342 | 11.053 | < 2.00E-16 | 3.148269456 | 2.527807987 | 3.921025893 |
| Statins | 0.70171 | 0.13251 | 5.295 | 1.19e-07 | 2.017201612 | 1.555795122 | 2.615448709 |
| APOE:Statins | -0.25282 | 0.14513 | -1.742 | 0.08150 | 0.776610176 | 0.584345316 | 1.032135193 |
| Sex(M):Statins | 0.08316 | 0.14570 | 0.571 | 0.56815 | 1.086715936 | 0.816767170 | 1.445885154 |
| Sex(M):APOE | -0.41877 | 0.14186 | -2.952 | 0.00316 | 0.657853211 | 0.498173116 | 0.868715780 |

**Figure 1** - Flow of participants through the study

**Figure 2** – Regression tree including APOE genotypes - The matched population was stratified on the basis of ApoE2 (n=10620, %=0.09), ApoE3 (n=80277, %=0.69) and ApoE4 (n=24121, %=0.21) genotypes.


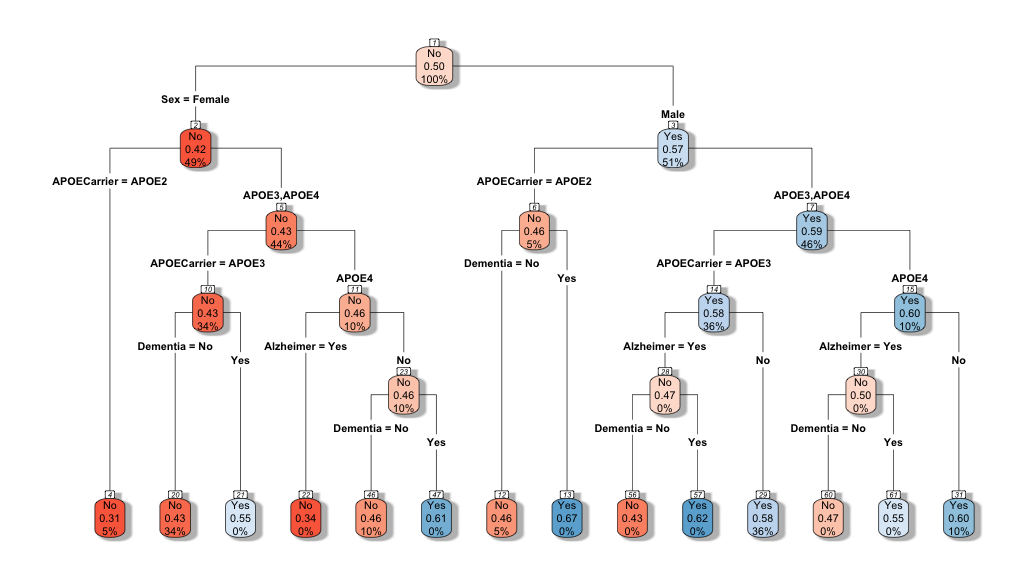


The regression tree indicates the strata for the disease of interest (dementia and AD) that should be considered among ApoE3 and ApoE4. It is interesting to note how the percentage of non-treated female and male ApoE2 subjects are comparable (5%).

**Figure 3:** Observed distributions (density plots) of reaction time at baseline, for males versus females (left) and ApoE carriers versus non-carriers (right).


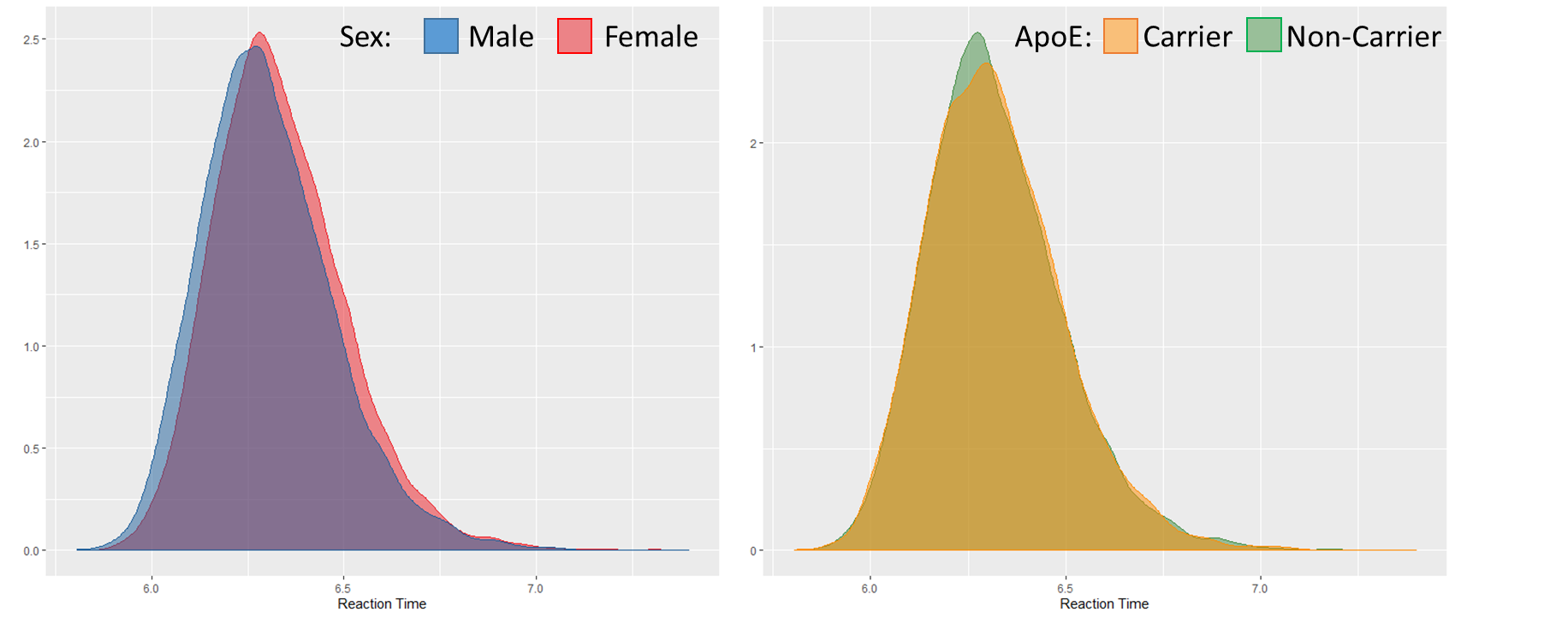


**Figure 4:** Hazard plot for the multivariate cox regression analysis. Full results are reported in table 3 of the main manuscript.


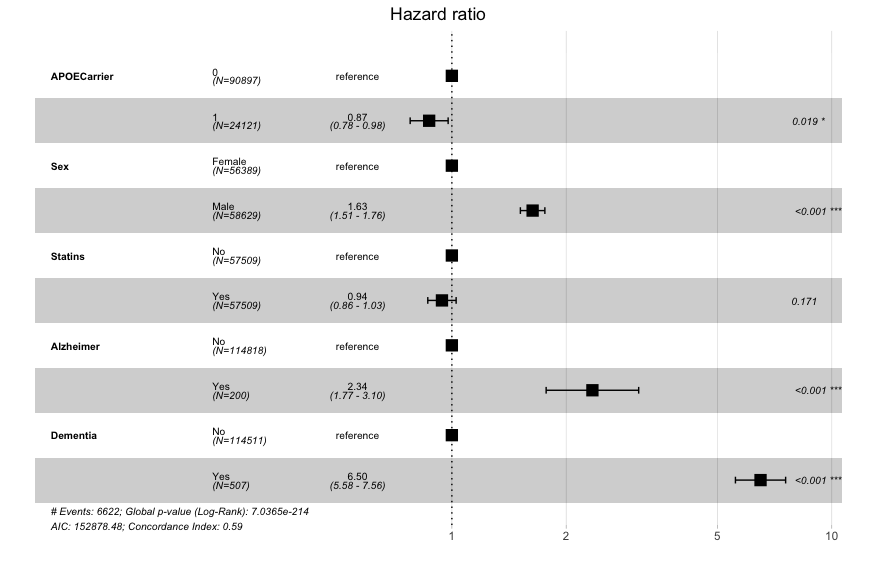


# Appendix C – Missingness mechanism

Determining from the observed data weather the missingness mechanics was MAR or MNAR, and proving it with a statistical evidence it would be impossible, unless managing to retrieve the missing data. Here some reason because we conjecture a MAR mechanism:

1. samples were retrieved for all the UK Biobank participant.
2. factors such as batch effect, time of the analyses might have an impact on missingness.
3. computing the APOE genotype form only one allele is impossible, thus we have marked as missing also subject with only one allele missing.
4. allele for which we don’t have data are reported as 00, thus it would be difficult to state id the sample was not yet analyzed or the experiment failed.
5. as indicated by Bycroft “for markers that failed at least one test in a given batch, we set the genotype calls in that batch to missing”.

Nevertheless, in the following table we report in variables of interest distributions in the two subgroups (ApoE missing and not missing).

|  | ApoE Non-Missing | ApoE Non-Missing |
| --- | --- | --- |
| N | 252327 | 55547 |
| Age At Recruitment (mean (SD)) | 62.06 (4.09) | 62.05 (4.05) |
| UniversityCollege = 1 (%) | 80513 (28.8) | 12297 (26.0) |
| Townsend deprivation index (mean (SD)) | -1.53 (2.97) | -1.32 (3.08) |
| Ethnicity (%) |  |  |
| AsianorAsianBritish | 4213 (1.5) | 247 (0.5) |
| BlackorBlackBritish | 3109 (1.1) | 141 (0.3) |
| Chinese | 641 (0.2) | 37 (0.1) |
| Donotknow | 115 (0.0) | 7 (0.0) |
| Mixed | 1083 (0.4) | 59 (0.1) |
| Otherethnicgroup | 1825 (0.7) | 108 (0.2) |
| Prefernottoanswer | 1006 (0.4) | 61 (0.1) |
| White | 267596 (95.7) | 46639 (98.6) |
| Sex = M (%) | 127716 (45.7) | 23671 (50.0) |
| Alzheimer = Yes (%) | 406 (0.1) | 64 (0.1) |
| Dementia = Yes (%) | 742 (0.3) | 123 (0.3) |
| Statins = Yes (%) | 61477 (22.0) | 11569 (24.5) |
| Reaction Test Mean Time (mean (SD)) | 581.85 (121.26) | 577.63 (116.95) |

# Appendix D – Propensity score matching results (Statins)

In the following we report the results of the matching process regarding Statin treatments. We report summary of mean differences in the matched cohorts for the considered variables, the estimated propensity score and the plots of the mean of each covariate against the estimated propensity score.

Summary of Balance for All Data:

|  | Means Treated | Means Control | Std. Mean Diff. | Var. Ratio | eCDF Mean | eCDF Max |
| --- | --- | --- | --- | --- | --- | --- |
| distance | 0.1360 | 0.1347 | 0.1023 | 10.387 | 0.0293 | 0.0474 |
| Ageatrecruitment | 619.896 | 623.871 | -0.0967 | 10.174 | 0.0221 | 0.0425 |
| Townsenddeprivationindexatrecruitment | -13.903 | -14.364 | 0.0151 | 10.202 | 0.0055 | 0.0159 |
| AgeCompletedEducation | 161.010 | 161.905 | -0.0266 | 10.080 | 0.0033 | 0.0233 |

Summary of Balance for Matched Data:

|  | Means Treated | Means Control | Std. Mean Diff. | Var. Ratio | eCDF Mean | eCDF Max | Std. Pair Dist. |
| --- | --- | --- | --- | --- | --- | --- | --- |
| distance | 0.1360 | 0.1360 | 0.0000 | 10.002 | 0.0000 | 0.0010 | 0.0001 |
| Ageatrecruitment | 619.896 | 619.677 | 0.0053 | 0.9976 | 0.0020 | 0.0059 | 0.2158 |
| Townsenddeprivationindexatrecruitment | -13.903 | -14.630 | 0.0238 | 10.213 | 0.0072 | 0.0161 | 0.8773 |
| AgeCompletedEducation | 161.010 | 161.380 | -0.0110 | 10.048 | 0.0020 | 0.0170 | 0.6230 |

Percent Balance Improvement:

|  | Std. Mean Diff. | Var. Ratio | eCDF Mean. | eCDF Ma. |
| --- | --- | --- | --- | --- |
| distance | 100.0 | 99.6 | 100 | 97.9 |
| Ageatrecruitment | 94.5 | 86.3 | 91 | 86.0 |
| Townsenddeprivationindexatrecruitment | -57.6 | -5.4 | -30 | -1.5 |
| AgeCompletedEducation | 58.6 | 39.3 | 38 | 27.1 |


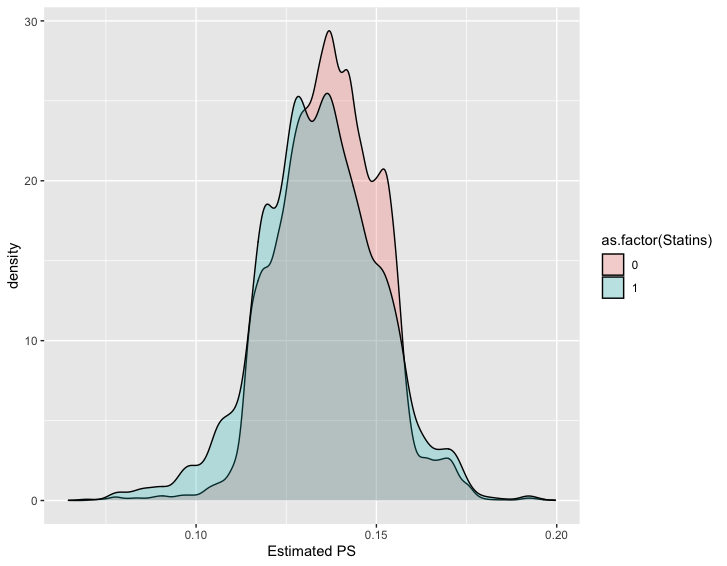


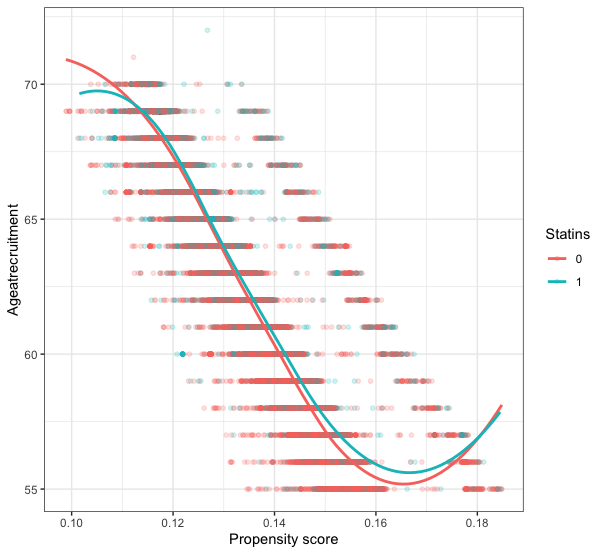

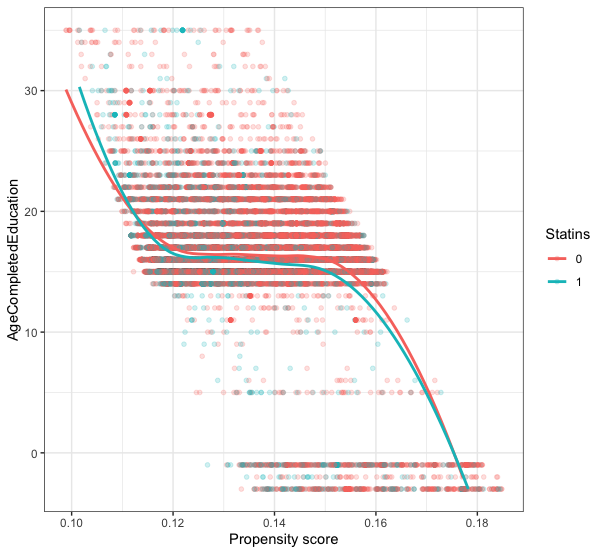


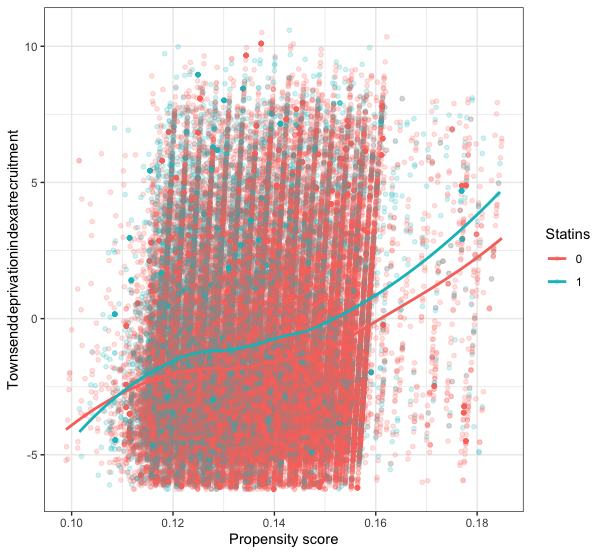

Supplement: Supplementary file 1 — Supporting information. [file TRC2-7-e12156-s001.docx]
